# Supplementary material for: CDK9 activity switch associated with AFF1 and HEXIM1 controls differentiation initiation from epidermal progenitors
Source: Nat Commun. 2022 Jul 29;13:4408. doi: 10.1038/s41467-022-32098-2 (PMC9338292; doi:10.1038/s41467-022-32098-2)
Supplement: Supplementary file 1 — Supplementary Information [file 41467_2022_32098_MOESM1_ESM.pdf]

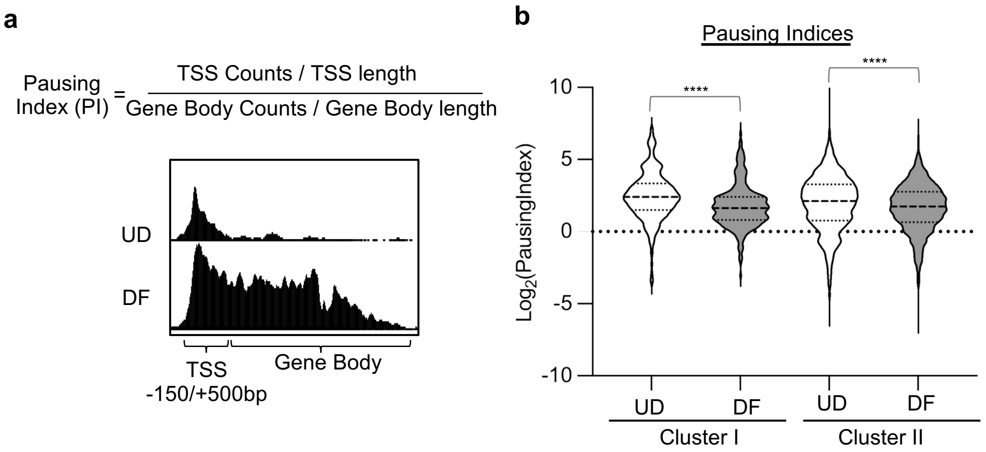


**Supplementary Fig. 1. KL treatment rapidly activates a subset of differentiation-upregulated genes with high Pol II enrichment.** (a) Illustration of Pausing Index calculation for Pol II pause release. Pausing indices were calculated by taking a ratio of total Pol II in promoter relative to total Pol II in gene bodies normalized by gene length. (b) Violin plot showing pausing indices in UD compared to DF in clusters I and II (****P < 0.0001, two-tailed, unpaired t-test). Source data are provided as a Source Data file.

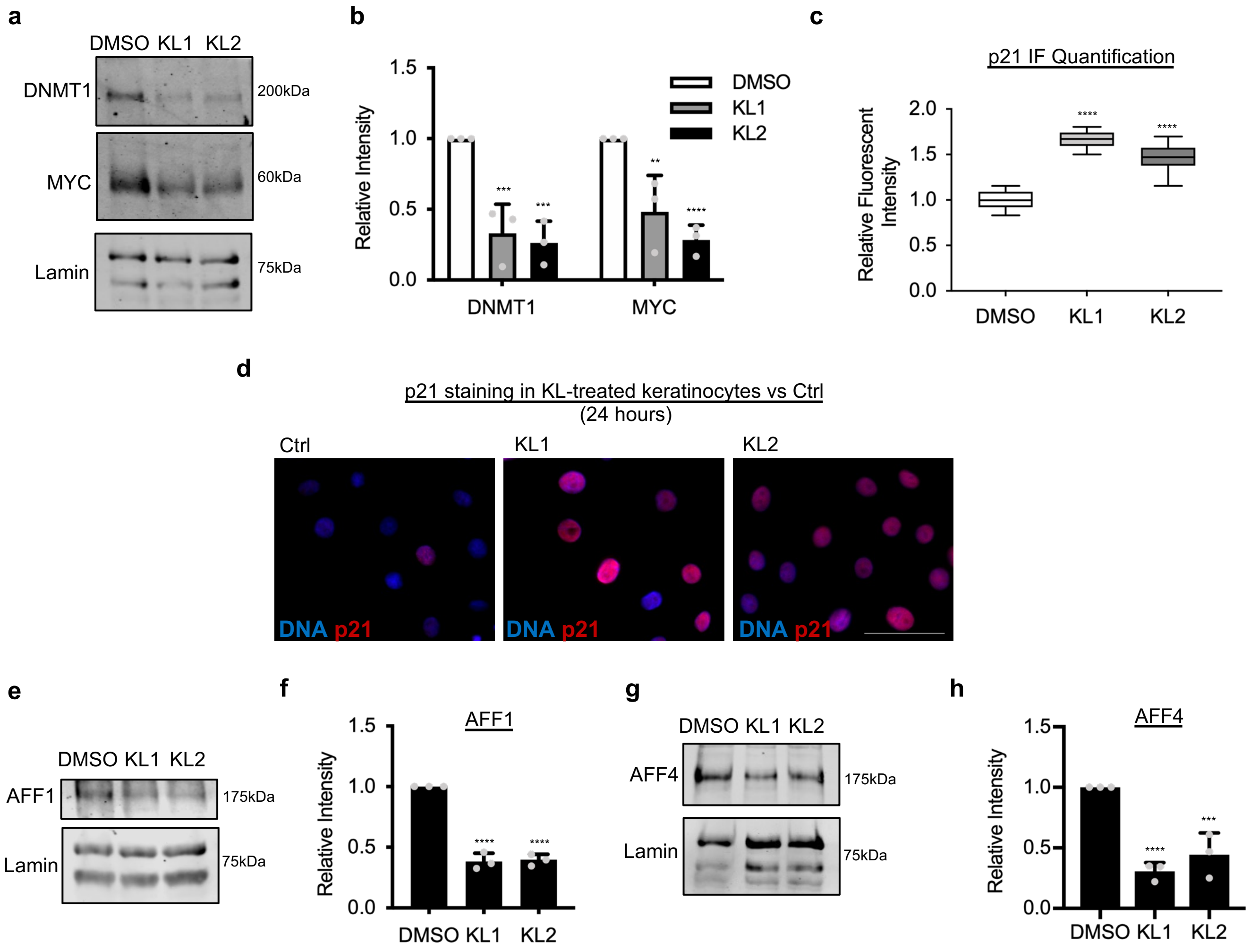


**Supplementary Fig. 2. KL treatment progressively upregulates differentiation-activating TF's and impairs proliferation.** (a,b) Western blot and quantification assessing progenitor marker genes, DNMT1 and MYC, with 24-hour KL treatment (scale = 50μm, n=3 biological replicates, ****P < 0.001, ***P<0.01, **P < 0.05, KL1 DNMT1 P = 0.0048, KL2 DNMT1 P = 0.0012, KL1 MYC P = 0.0250, KL2 MYC P = 0.0003, two-tailed, unpaired t-test, data are presented as mean values +/- standard deviation). (c,d) p21 Immunofluorescent staining and quantification in control, KL1, or KL2 treated keratinocytes (n=15 images quantified for each of 3 biological replicates, 1 representative shown,****P < 0.0001, two-tailed, unpaired t-test, box plot represents first through third quartiles, minima, and maxima). (e-h) Western blots and quantifications showing reduced protein level of AFF1 and AFF4 with 24-hour KL treatment (n=3 biological replicates, ****P < 0.001, ***P < 0.01, AFF1 KL1 P < 0.0001, AFF1 KL2 P < 0.0001 , AFF4 KL1 P < 0.0001, AFF4 KL2 P = 0.0060, two-tailed, unpaired t-test, data are presented as mean values +/- standard deviation). Source data are provided as a Source Data file.

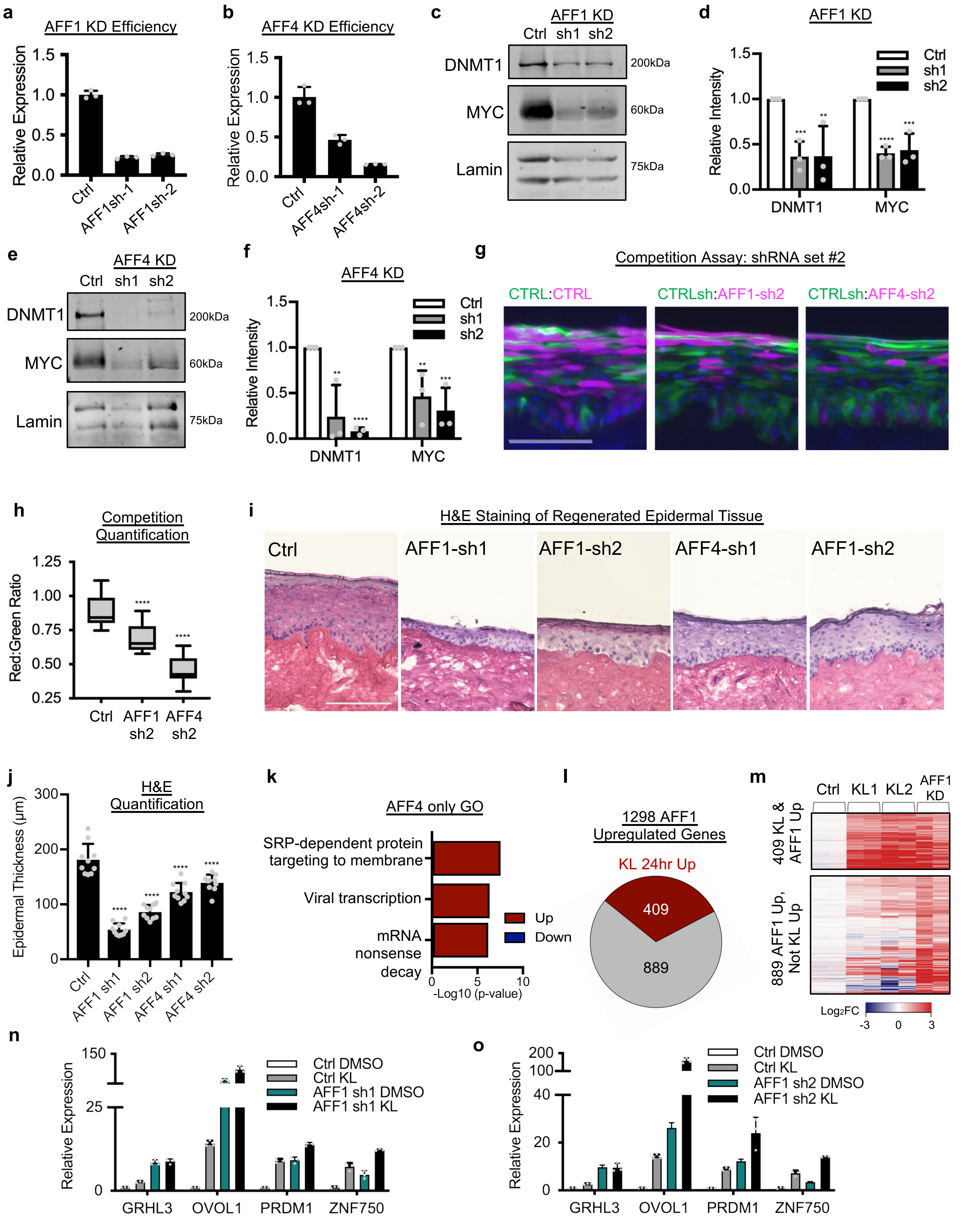

a
AFF1 KD Efficiency
Relative Expression
1.5
1.0
0.5
0.0
Ctrl
AFF1sh-1
AFF1sh-2
b
AFF4 KD Efficiency
Relative Expression
Ctrl
AFF4sh-1
AFF4sh-2
c
AFF1 KD
Ctrl sh1 sh2
DNMT1
MYC
Lamin
200kDa
60kDa
75kDa
d
AFF1 KD
Relative Intensity
Ctrl
sh1
sh2
DNMT1
MYC
e
AFF4 KD
Ctrl sh1 sh2
DNMT1
MYC
Lamin
200kDa
60kDa
75kDa
f
AFF4 KD
Relative Intensity
Ctrl
sh1
sh2
DNMT1
MYC
g
Competition Assay: shRNA set #2
CTRL:CTRL
CTRLsh:AFF1-sh2
CTRLsh:AFF4-sh2
h
Competition Quantification
Red:Green Ratio
1.25
1.00
0.75
0.50
0.25
Ctrl
AFF1 sh2
AFF4 sh2
i
H&E Staining of Regenerated Epidermal Tissue
Ctrl
AFF1-sh1
AFF1-sh2
AFF4-sh1
AFF1-sh2
j
H&E Quantification
Epidermal Thickness (μm)
300
200
100
0
Ctrl
AFF1 sh1
AFF1 sh2
AFF4 sh1
AFF4 sh2
k
AFF4 only GO
SRP-dependent protein targeting to membrane
Viral transcription
mRNA nonsense decay
Up
Down
-Log10 (p-value)
0
5
10
l
1298 AFF1 Upregulated Genes
KL 24hr Up
409
889
m
Ctrl
KL1
KL2
AFF1 KD
409 KL & AFF1 Up
889 AFF1 Up, Not KL Up
Log2FC
-3
0
3
n
Relative Expression
150
25
0
Ctrl DMSO
Ctrl KL
AFF1 sh1 DMSO
AFF1 sh1 KL
GRHL3
OVOL1
PRDM1
ZNF750
o
Relative Expression
200
100
40
20
0
Ctrl DMSO
Ctrl KL
AFF1 sh2 DMSO
AFF1 sh2 KL
GRHL3
OVOL1
PRDM1
ZNF750

**Supplementary Fig. 3. SEC scaffold, AFF1, but not AFF4, is essential for repressing epidermal differentiation.** (**a,b**) qRT-PCR showing knockdown (KD) efficiency of shRNA's targeting AFF1 or AFF4 (n = 3 technical replicates, data are presented as mean values +/- standard deviation). (**c-f**) Western blot and quantification assessing progenitor markers, DNMT1 and MYC, with AFF1 or AFF4 knockdown (n=3 biological replicates, ****P < 0.001, ***P <0.01, **P < 0.05, AFF1 sh1 DNMT1 P = 0.0027 , AFF1 sh2 DNMT1 P = 0.0301, AFF1 sh1 MYC P < 0.0001 , AFF1 sh2 MYC P = 0.0058, AFF4 sh1 DNMT1 P = 0.0191, AFF4 sh2 DNMT1 P < 0.0001 , AFF4 sh1 MYC P = 0.0302, AFF4 sh2 MYC P = 0.0087, two-tailed, unpaired t-test, data are presented as mean values +/- standard deviation). (**g,h)** Representative images and red:green fluorescence-quantification of epidermal tissue sections from competition assay with shRNA 2 for AFF1 or AFF4 relative to non-targeting control. Red labeled cells are represented in magenta. (scale bar = 125μm, n = 10 images from biological replicate 1 of 3, ****P < 0.001, AFF1 P = 0.0008, AFF4 P < 0.0001, two-tailed, unpaired t-test, box plot represents first through third quartiles, minima, and maxima). (**i,j)** H&E staining and quantification of epidermis regenerated in organotypic culture with AFF1 or AFF4 knockdown cells relative to non-targeting control (scale bar = 200μm, n = 10 images, ****P < 0.001, AFF1 sh1, AFF1 sh2 and AFF4 sh1 P < 0.0001, AFF4 sh2 P = 0.0007, two-tailed, unpaired t-test, data are presented as mean values +/- standard deviation). (**k**) Top Gene Ontology (GO) terms of differentially-expressed genes uniquely in AFF4 KD RNA-seq, but not in AFF1 KD RNA-seq (two-tailed, Fisher's exact test). (**l**) Pie chart showing total number of AFF1 upregulated genes also upregulated with 24-hour KL treatment. (**m**) Heatmap showing expression changes relative to control at genes upregulated by both AFF1 knockdown and 24-hour KL and at genes upregulated by AFF1 knockdown but not significantly upregulated by 24-hour KL treatment. (**n,o**) Summation of the qRT-PCR data as shown in Fig.3q-s, comparing mRNA levels of differentiation activating TFs between DMSO control and KL2 treatment with non-targeting control or AFF1 knockdown using two independent shRNA's where everything is relative to non-targeting, DMSO treated control condition (n =3 technical replicates, data are presented as mean values +/- standard deviation). Source data are provided as a Source Data file.

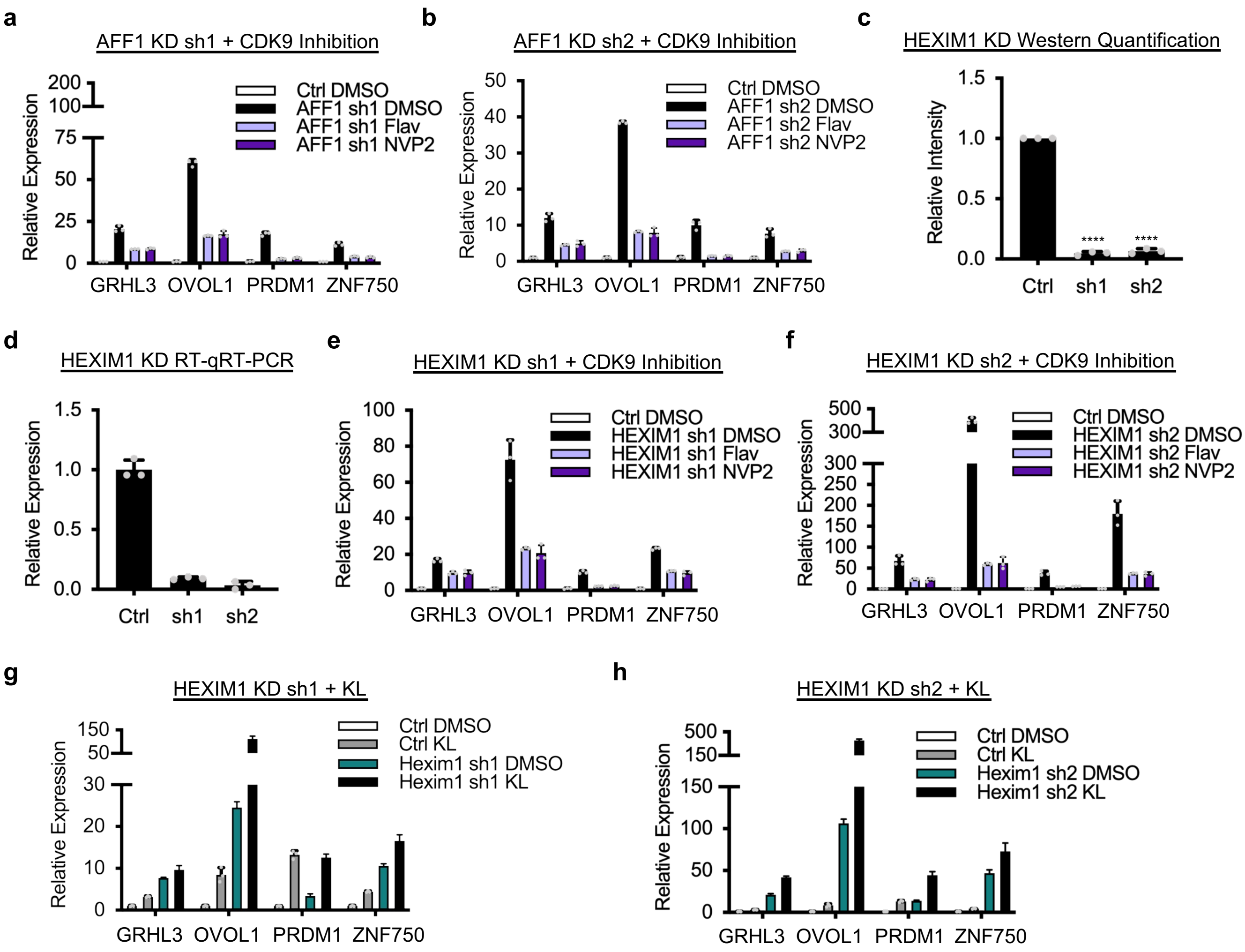


**Supplementary Fig. 4. AFF1 associates with inactive PTEFb to repress target gene expression.** (**a**,**b**) qRT-PCR showing expression of differentiation activators with AFF1 knockdown alone or in combination with CDK9 inhibitors, flavopiridol (Flav) or NVP2, relative to non-targeting, DMSO control (n=3, data are presented as mean values +/- standard deviation). (**c**) Quantification of western blot showing HEXIM1 knockdown efficiency (Fig. 4c) (n = 3 biological replicates, ****P < 0.0001, two-tailed, unpaired t-test, data are presented as mean values +/- standard deviation). (**d)** qRT-PCR showing the knockdown efficiency of shRNA's targeting HEXIM1 relative to the non-target control shRNA (n = 3 technical replicates, two-tailed, unpaired t-test, data are presented as mean values +/- standard deviation). (**e**,**f**) qRT-PCR showing expression of differentiation activators with HEXIM1 knockdown with DMSO control or in combination with CDK9 inhibitors, flavopiridol (Flav) or NVP2, relative to non-targeting, DMSO control (n=3 technical replicates, data are presented as mean values +/- standard deviation). (**g**,**h**) Summation of the qRT-PCR data as shown in Fig.4g-i, comparing mRNA levels of differentiation activating TFs between DMSO control and KL2 treatment with non-targeting control or HEXIM1 knockdown using two independent shRNA's where everything is relative to the non-targeting, DMSO treatment control condition (n =3 technical replicates, data are presented as mean values +/- standard deviation). Source data are provided as a Source Data file.

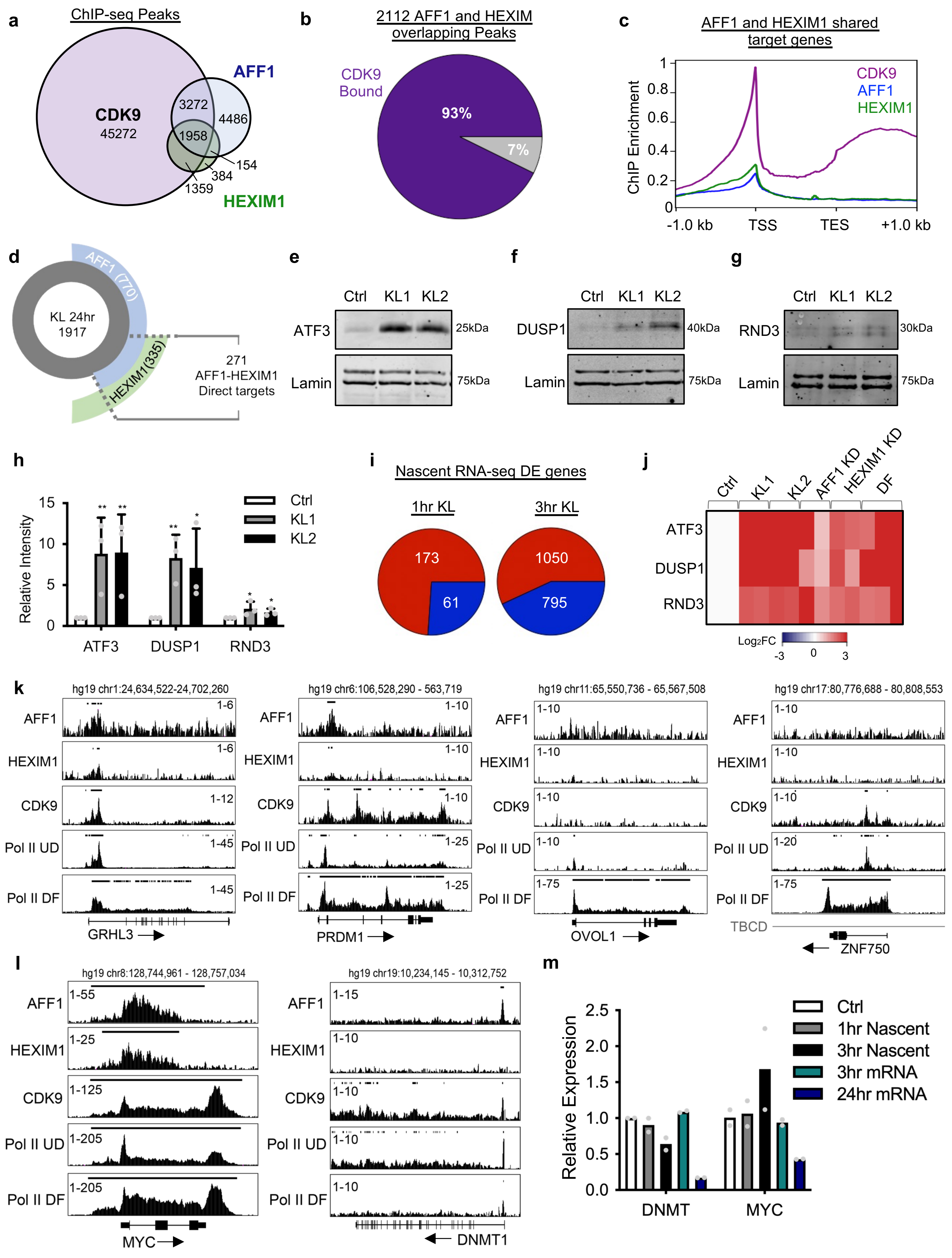

a
ChIP-seq Peaks
CDK9
45272
AFF1
3272
4486
1958
154
384
1359
HEXIM1
b
2112 AFF1 and HEXIM overlapping Peaks
CDK9 Bound
93%
7%
c
AFF1 and HEXIM1 shared target genes
ChIP Enrichment
CDK9
AFF1
HEXIM1
-1.0 kb
TSS
TES
+1.0 kb
d
AFF1 (770)
KL 24hr 1917
HEXIM1(335)
271 AFF1-HEXIM1 Direct targets
e
Ctrl KL1 KL2
ATF3
25kDa
Lamin
75kDa
f
Ctrl KL1 KL2
DUSP1
40kDa
Lamin
75kDa
g
Ctrl KL1 KL2
RND3
30kDa
Lamin
75kDa
h
Relative Intensity
Ctrl
KL1
KL2
ATF3
DUSP1
RND3
i
Nascent RNA-seq DE genes
1hr KL
3hr KL
173
61
1050
795
j
Ctrl
KL1
KL2
AFF1 KD
HEXIM1 KD
DF
ATF3
DUSP1
RND3
Log2FC
k
hg19 chr1:24,634,522-24,702,260
hg19 chr6:106,528,290 - 563,719
hg19 chr11:65,550,736 - 65,567,508
hg19 chr17:80,776,688 - 80,808,553
AFF1
HEXIM1
CDK9
Pol II UD
Pol II DF
GRHL3
PRDM1
OVOL1
TBCD
ZNF750
l
hg19 chr8:128,744,961 - 128,757,034
hg19 chr19:10,234,145 - 10,312,752
AFF1
HEXIM1
CDK9
Pol II UD
Pol II DF
MYC
DNMT1
m
Relative Expression
Ctrl
1hr Nascent
3hr Nascent
3hr mRNA
24hr mRNA
DNMT
MYC

**Supplementary Fig. 5. HEXIM1 and AFF1 colocalize to directly suppress rapid-response targets.** (**a**) Venn diagram comparing AFF1, HEXIM1, and CDK9 ChIP-seq peaks in progenitors. (**b**) Pie chart showing percentage of AFF1-HEXIM1 overlapping peaks co-occupied by CDK9. (**c**) Average-profile plot showing AFF1, HEXIM1, and CDK9 ChIP-seq enrichment at AFF1-HEXIM1 shared target genes. (**d**) Shared target genes among AFF1 ChIP-seq, HEXIM1 ChIP-seq and the differentially expressed genes in keratinocytes with 24-hr KL treatment. **(e-h**) Western blots and quantification showing ATF3, DUSP1, and RND3 upregulated at the protein level with 3-hour KL treatment (n=3 biological replicates, $^{**}P < 0.05$, $^{*}P < 0.1$, ATF3 KL1 $P = 0.0364$, ATF3 KL2 $P = 0.0410$, DUSP1 KL1 $P = 0.0113$, DUSP1 KL2 $P = 0.0899$, RND3 KL1 = 0.0917, RND3 KL2 = 0.0610, two-tailed, unpaired t-test, data are presented as mean values +/- standard deviation). (**i**) Pie charts showing percentage of differentially expressed genes from 1 and 3-hour nascent RNA-seq that are up or downregulated. (**j**) Heatmap showing the relative fold change of ATF3, DUSP1, and RND3 in keratinocytes with KL treatment (3 hours), AFF1 knockdown (KD), HEXIM1 KD, or differentiation (DF), based on our RNA-seq data. (**k**) Genome browser tracks showing the enrichment of AFF1, HEXIM1, CDK9, undifferentiated (UD) Pol II, and differentiated (DF) Pol II ChIP-seq signal at differentiation activating genes. (**l**) Genome browser tracks showing the enrichment of AFF1, HEXIM1, CDK9, undifferentiated (UD) Pol II, and differentiated (DF) Pol II ChIP-seq signal at self-renewal genes, MYC and DNMT1. (**m**) Expression of DNMT1 and MYC relative to control from Nascent or mRNA-seq (n=2 technical replicates). Source data are provided as a Source Data file.

**a**

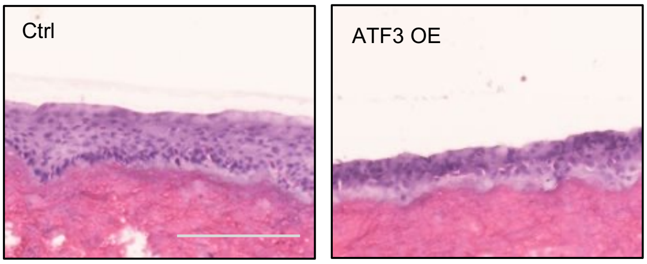


**b**

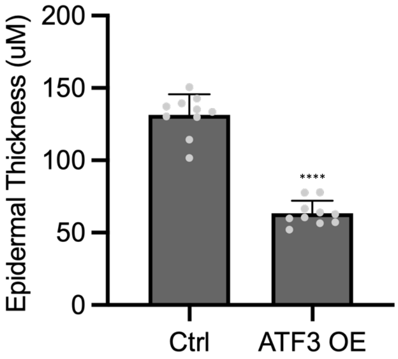


**Supplementary Fig. 6. Expression of SEC rapid-response gene ATF3 drives keratinocyte differentiation.** (**a,b**) H&E staining and thickness quantification of epidermis regenerated in organotypic culture with control or ATF3 overexpression (scale = 100μm, n=10 images from 1 of 3 biological replicates, ****P < 0.0001, two-tailed, unpaired t-test, data are presented as mean values +/- standard deviation). Source data are provided as a Source Data file.

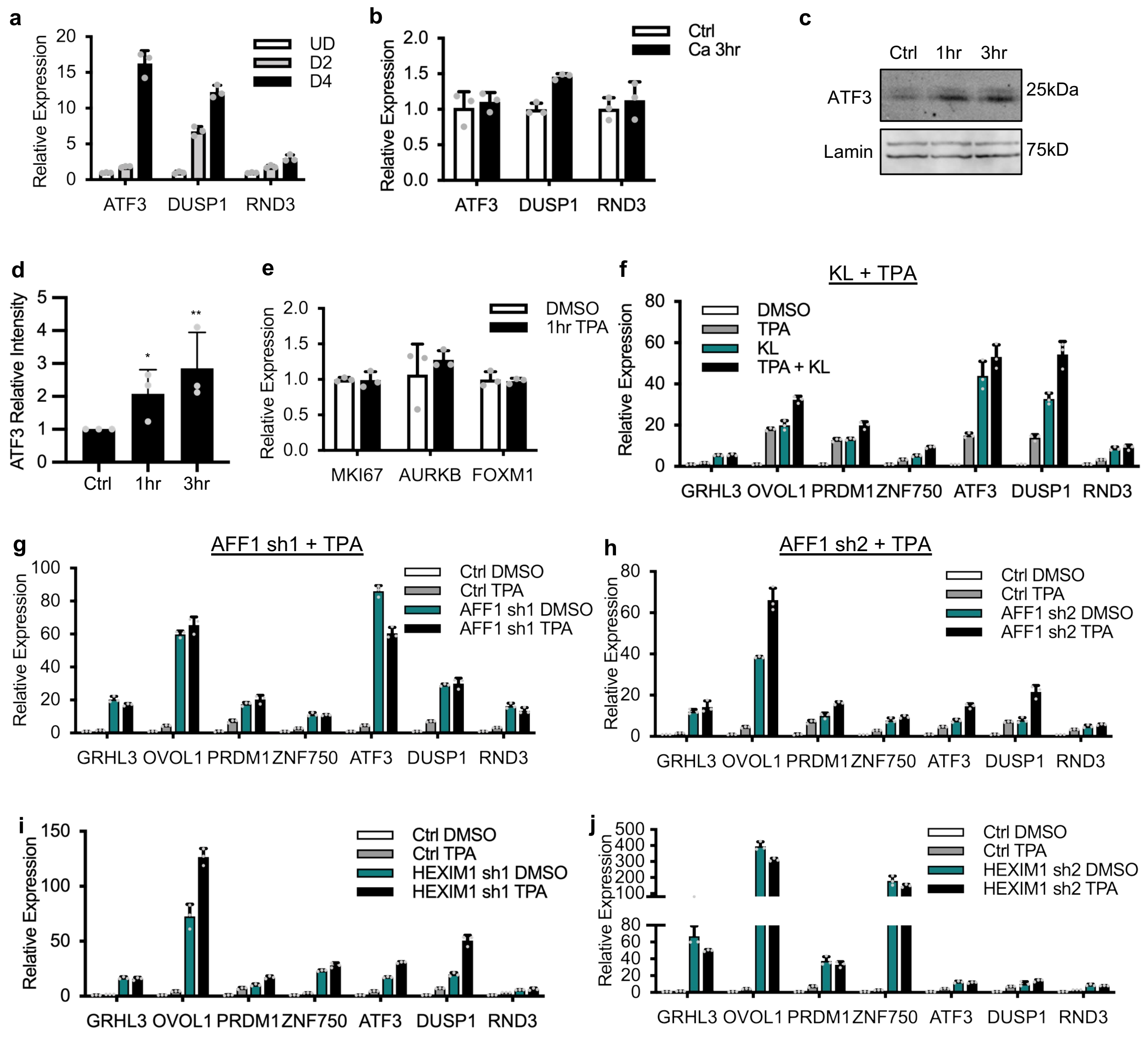


**Supplementary Fig. 7. SEC mediates the rapid upregulation of differentiation in response to PKC signaling.** (**a,b**) qRT-PCR showing the relative mRNA expression of ATF3, DUSP1, and RND3 in keratinocytes cultured in high $Ca^{2+}$ (1.2mM), 100% confluency condition, in longer-term, two (D2) or four (D4) days or short-term, sub-confluent, three hours (Ca 3hr), conditions (n = 3 technical replicates, data are presented as mean values +/- standard deviation). (**c,d**) Western blot and quantification showing sustained ATF3 protein level increase with 1-hr or 3-hr TPA treatment (n=3 biological replicates, **P < 0.05, *P < 0.1, 1hr P = 0.0657, 3hr P = 0.0426, two-tailed, unpaired t-test, data are presented as mean values +/- standard deviation). (**e**) qRT-PCR showing mRNA levels of proliferation markers with 1-hour TPA relative to DMSO control (n = 3 technical replicates, data are presented as mean values +/- standard deviation). (**f**) Summation of Fig. 7c,d, qRT-PCR showing gene expression changes with TPA, KL, or TPA in combination with KL all relative to DMSO control (n=3 technical replicates, data are presented as mean values +/- standard deviation). **(g-j**) qRT-PCR showing gene expression changes with TPA in addition to AFF1 or HEXIM1 knockdown relative to a non-targeting DMSO control (n=3 technical replicates, data are presented as mean values +/- standard deviation). Source data are provided as a Source Data file.

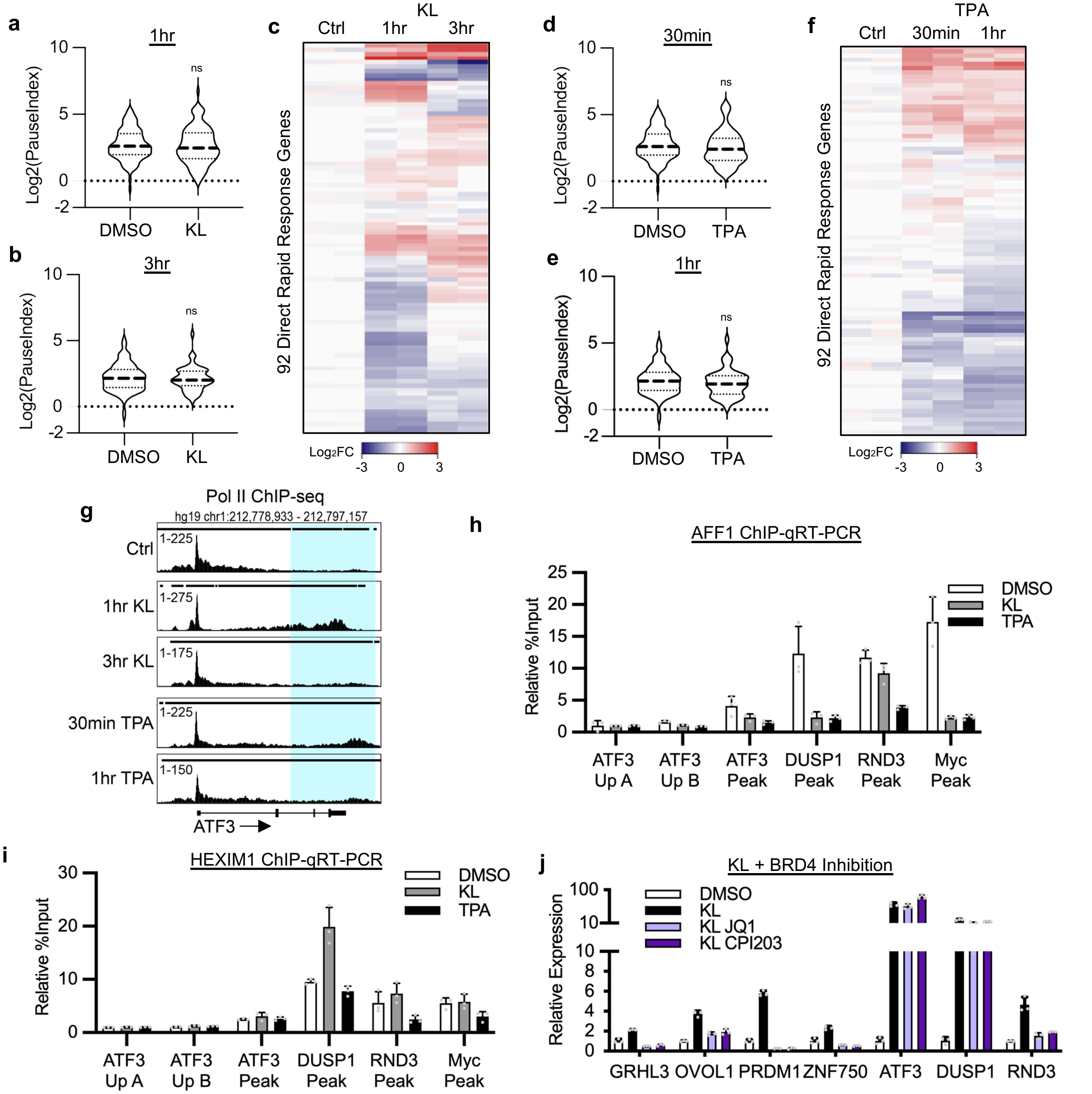


**Supplementary Fig. 8. CDK9 dissociation from repressive, HEXIM1 rapidly induces gene expression.** (**a,b**) Pol II Pausing Index distribution with 1 or 3-hr KL treatment at 92 rapid-response genes (ns = not significant, 1hr P = 0.7980, 3hr P = 0.7749 ,two-tailed, unpaired t-test). (**c**) Heatmap showing dynamic pausing indices at 92 rapid-response genes with 1 or 3-hour KL treatment. (**d,e**) Pol II Pausing Index distribution with 30min or 1-hr TPA treatment at 92 rapid-response genes (ns = not significant, 30min P = 0.2824, 1hr P = 0.2065, two-tailed, unpaired t-test). (**f**) Heatmap showing dynamic pausing indices at 92 rapid-response genes with 30min or 1-hr TPA treatment. (**g**) Genome browser tracks showing Pol II ChIP-seq enrichment with DMSO control (Ctrl), 1 or 3-hour KL, and 30min or 1-hour TPA treatment at ATF3. (**h,i**) AFF1 and HEXIM1 ChIP-qRT-PCR showing percent input of KL or TPA conditions relative to DMSO control at upstream control locations and at known peak locations (n=3 technical replicates, data are presented as mean values +/- standard deviation). (**j**) qRT-PCR showing changes in gene expression with KL treatment alone or in combination with BRD4 inhibitors, JQ1 or CPI203, relative to a DMSO control (n=3 technical replicates, data are presented as mean values +/- standard deviation). Source data are provided as a Source Data file.
