# Supplementary material for: CDK9 activity switch associated with AFF1 and HEXIM1 controls differentiation initiation from epidermal progenitors
Source: Nat Commun. 2022 Jul 29;13:4408. doi: 10.1038/s41467-022-32098-2 (PMC9338292; doi:10.1038/s41467-022-32098-2)
Supplement: Supplementary file 3 — Description of Additional Supplementary Files [file 41467_2022_32098_MOESM3_ESM.pdf]

Description of Additional Supplementary Files

File Name: Supplementary Data 1
Description: Clusters of genes differentially expressed during epidermal differentiation.

File Name: Supplementary Data 2
Description: Genes differentially expressed with 3-hour KL treatment.

File Name: Supplementary Data 3
Description: Genes differentially expressed with 24-hour KL treatment.

File Name: Supplementary Data 4
Description: Genes differentially expressed with AFF1 or AFF4 knockdown.

File Name: Supplementary Data 5
Description: Genes differentially expressed with HEXIM1 knockdown.

File Name: Supplementary Data 6
Description: 92 direct, rapid-response SEC targets.

File Name: Supplementary Data 7
Description: Genes differentially expressed with 1-hour TPA treatment.

File Name: Supplementary Data 8
Description: Oligonucleotides used for qRT-qPCR.
