# Supplementary material for: CDK9 activity switch associated with AFF1 and HEXIM1 controls differentiation initiation from epidermal progenitors
Source: Nat Commun. 2022 Jul 29;13:4408. doi: 10.1038/s41467-022-32098-2 (PMC9338292; doi:10.1038/s41467-022-32098-2)
Supplement: Supplementary file 12 — Reporting Summary [file 41467_2022_32098_MOESM12_ESM.pdf]

nature research

Corresponding author(s): Xiaomin Bao

Last updated by author(s): Jul 1, 2022

Reporting Summary

Nature Research wishes to improve the reproducibility of the work that we publish. This form provides structure for consistency and transparency in reporting. For further information on Nature Research policies, see our Editorial Policies and the Editorial Policy Checklist.

Statistics

For all statistical analyses, confirm that the following items are present in the figure legend, table legend, main text, or Methods section.

n/a	Confirmed	
☐	☒	The exact sample size (*n*) for each experimental group/condition, given as a discrete number and unit of measurement
☐	☒	A statement on whether measurements were taken from distinct samples or whether the same sample was measured repeatedly
☐	☒	The statistical test(s) used AND whether they are one- or two-sided<br>*Only common tests should be described solely by name; describe more complex techniques in the Methods section.*
☐	☒	A description of all covariates tested
☐	☒	A description of any assumptions or corrections, such as tests of normality and adjustment for multiple comparisons
☐	☒	A full description of the statistical parameters including central tendency (e.g. means) or other basic estimates (e.g. regression coefficient) AND variation (e.g. standard deviation) or associated estimates of uncertainty (e.g. confidence intervals)
☐	☒	For null hypothesis testing, the test statistic (e.g. *F*, *t*, *r*) with confidence intervals, effect sizes, degrees of freedom and *P* value noted<br>*Give P values as exact values whenever suitable.*
☒	☐	For Bayesian analysis, information on the choice of priors and Markov chain Monte Carlo settings
☒	☐	For hierarchical and complex designs, identification of the appropriate level for tests and full reporting of outcomes
☒	☐	Estimates of effect sizes (e.g. Cohen's *d*, Pearson's *r*), indicating how they were calculated

Our web collection on statistics for biologists contains articles on many of the points above.

Software and code

Policy information about availability of computer code

Data collection	Immunoblot : Image Studio Software version 5.2 (LI-COR)<br>qPCR: QuantStudio Design & Analysis Software 1.3.1 (Thermo Fisher)<br>Immunofluorescence: EVOS FL Auto 2 Imaging System Software Revision 2.0.1732.0 (Thermo Fisher)
Data analysis	This paper does not report original code.<br>Immunoblot analysis: Image Studio Software version 5.2 (LI-COR)<br>qPCR analysis: GraphPad Prism version 7<br>Microscopy image processing: ImageJ (NIH) version 2.1.0, Python 3 with Scikit-image package<br>For RNA-seq and ChIP-seq, the softwares used are described in full in the material and methods section, including R Studio version 3.6.2, Bowtie2, MACS2, BEDtools, deepTools, ggplot2, EdgeR, HISAT2, SAMtools, HTSeq, DESeq2m and DAVID Gene Ontology Analysis tool

For manuscripts utilizing custom algorithms or software that are central to the research but not yet described in published literature, software must be made available to editors and reviewers. We strongly encourage code deposition in a community repository (e.g. GitHub). See the Nature Research guidelines for submitting code & software for further information.

Data

Policy information about availability of data

All manuscripts must include a data availability statement. This statement should provide the following information, where applicable:

- Accession codes, unique identifiers, or web links for publicly available datasets
- A list of figures that have associated raw data
- A description of any restrictions on data availability

RNA-seq and ChIP-seq data have been deposited at GEO (GSE#182959) and are publicly available. Raw data associated with all the figures are uploaded as an excel

file. Any additional information required to reanalyze the data reported in this paper is available from the lead contact upon request. Source data are provided with this paper as Source Data file.

Field-specific reporting

Please select the one below that is the best fit for your research. If you are not sure, read the appropriate sections before making your selection.

☒ Life sciences ☐ Behavioural & social sciences ☐ Ecological, evolutionary & environmental sciences

For a reference copy of the document with all sections, see nature.com/documents/nr-reporting-summary-flat.pdf

Life sciences study design

All studies must disclose on these points even when the disclosure is negative.

Sample size	For all experiments, keratinocytes from at least three donors were combined as is standard for primary keratinocyte cell culture (Bao et al. 2017, Chen et al. 2021). For knockdown RNA-seq experiments, two non-targeting control and two different shRNA's were used as was done in Bao et al. 2017. For ChIP-seq, two biological replicates were completed for each condition (Li et al. 2021). qRT-PCR experiments shown were performed with three technical replicates (Sen et al. 2010). These qRT-PCR's were further validated by an additional 1-2 biological replicates performed either in technical duplicate or triplicate as well as by RNA-seq in the case of knockdown experiments. For all tissue-based experiments, three organotypic cultures were generated for each condition (Li et al. 2021). To ensure robust quantification, at least ten images were taken per replicate. One representative biological replicate is shown, but all three replicates were quantified and demonstrated a consistent trend. For immunofluorescence, three biological replicates were performed and quantified. As with histological experiments, at least ten images were taken per sample. All replicates showed the same trend; one representative image is shown.
Data exclusions	No data were excluded from the analyses.
Replication	All experiments were performed in either technical or biological replicates. All replicates were used in the preparation of each data figure.
Randomization	Randomization was not necessary as experiments were carefully controlled. Primary human keratinocytes from at least three independent de-identified donors were randomly mixed, and these cells were treated the same across conditions.
Blinding	Given the quantitative nature of all experimental results, blinding was not required. No subjective data interpretation or analysis was applied.

Reporting for specific materials, systems and methods

We require information from authors about some types of materials, experimental systems and methods used in many studies. Here, indicate whether each material, system or method listed is relevant to your study. If you are not sure if a list item applies to your research, read the appropriate section before selecting a response.

Materials & experimental systems

n/a	Involved in the study	
☐	☒	Antibodies
☐	☒	Eukaryotic cell lines
☒	☐	Palaeontology and archaeology
☒	☐	Animals and other organisms
☐	☒	Human research participants
☒	☐	Clinical data
☒	☐	Dual use research of concern

Methods

n/a	Involved in the study	
☐	☒	ChIP-seq
☒	☐	Flow cytometry
☒	☐	MRI-based neuroimaging

Antibodies

Antibodies used

Mouse monoclonal anti-Ki67 (clone number not available) Santa Cruz Cat#sc-23900; RRID:AB_627859 (1:50)
Mouse monoclonal anti-Lamin A/C (E1) Santa Cruz Cat#sc-376248; RRID:AB_10991536 (1:1000)
Rabbit polyclonal anti-AFF1 Bethyl A-302-344A-T; RRID:AB_1850255 (1:500)
Rabbit anti-AFF1 serum, (Lin et al., 2011), Ali Shilatifard Lab (5µL per ChIP experiment)
Rabbit polyclonal anti-AFF4 Abclonal Cat#A4644; RRID:AB_2765799 (1:1000)
Rabbit polyclonal anti-HEXIM1 Bethyl Cat#A303-112A-T; RRID:AB_10895441 (1:1000)
Rabbit polyclonal anti-HEXIM1 Abcam Cat#ab25388; RRID:AB_2233058 (3µL per ChIP experiment)
Rabbit monoclonal anti-ATF-3 (E9J4N) Cell Signaling Cat#18665; RRID:AB_2827506 (1:1000)
Rabbit monoclonal anti-DUSP1 (E8L7D) Cell Signaling Cat#48625; RRID:AB_2893127 (1:1000)
Mouse monoclonal anti-RhoE (RND3) (4) Cell Signaling Cat#3664; RRID:AB_2181841 (1:300)
Rabbit monoclonal anti-RNA Polymerase II (D8L4Y), Cell Signaling #14958; RRID:AB_2687876 (1.5µL per ChIP experiment)
Mouse monoclonal anti-HA (66006-2-Ig), Protein Tech Cat#66006; RRID:AB_2857911 (6µL per ChIP experiment)
Rabbit monoclonal anti-p21 (12D1) , Cell Signaling #2947; RRID:AB_823586 (1:200)
Rabbit monoclonal anti-MYC (D84C12), Cell Signaling #5605; RRID:AB_1903938 (1:1000)

	Rabbit monoclonal anti-DNMT1 (D63A6), Cell Signaling #5032; RRID:AB_10548197 (1:1000)<br>Rabbit monoclonal anti-HA (C29F4), Cell Signaling #3724; RRID:AB_1549585 (1:1000)<br>Rabbit monoclonal anti-CDK9 (C12F7), Cell Signaling #2316; RRID:AB_2291505 (1:1000)<br>Rabbit polyclonal anti-PhosphoThr186 CDK9, Cell Signaling #2549; RRID:AB_2077300 (1:1000)<br>Mouse monoclonal anti-IGG (G3A1), Cell Signaling #5415; RRID:AB_10829607 (1.2µL per ChIP experiment)<br>Goat anti-mouse, secondary for western, Li-COR, #926-68020; RRID AB_10706161 (1:20,000)<br>Goat anti-rabbit, secondary for western, Li-COR #926-32211; RRID AB_621843 (1:20,000)<br>Goat anti-mouse 594, secondary for immunofluorescence staining, Thermo Fisher, #A11005, (1:400)<br>Goat anti-rabbit 488, secondary for immunofluorescence staining, Thermo Fisher, #A11034, (1:400)
Validation	Mouse monoclonal anti-Ki67 was validated for immunofluorescence staining in a human cell line. https://datasheets.scbt.com/sc-23900.pdf<br>Mouse monoclonal anti-Lamin A/C (E1) was validated for western blotting in a human cell line. https://www.scbt.com/p/lamin-a-c-antibody-e-1<br>Rabbit polyclonal anti-AFF1 Bethyl A-302-344A-T was validated for western blotting in a human cell line. https://www.fortislife.com/products/primary-antibodies/rabbit-anti-af4-antibody/BETHYL-A302-344<br>Rabbit anti-AFF1 serum for ChIP-seq was validated by the Shilatifard lab and published in Lin et al. 2011.<br>Rabbit polyclonal anti-AFF4 Abclonal was validated for western blotting in human cell lines. https://abclonal.com/Datasheet/Antibodies/A4644.pdf<br>Rabbit polyclonal anti-HEXIM1 Bethyl was validated for western blotting in a human cell line. https://www.fortislife.com/products/primary-antibodies/rabbit-anti-hexim1-antibody/BETHYL-A303-112<br>Rabbit polyclonal anti-HEXIM1 Abcam was validated for immunoprecipitation in human cell line. https://www.abcam.com/hexim1-antibody-ab25388.html?productWallTab=ShowAll<br>Rabbit monoclonal anti-ATF-3 (E9J4N) Cell Signaling was validated in human cell lines. https://www.cellsignal.com/products/primary-antibodies/atf-3-e9j4n-rabbit-mab/18665<br>Rabbit monoclonal anti-DUSP1 (E8L7D) Cell Signaling was validated for western blotting in human cell line. https://www.cellsignal.com/products/primary-antibodies/dusp1-mkp1-e8l7d-rabbit-mab/48625<br>Mouse monoclonal anti-RhoE (RND3) (4) Cell Signaling was validated for western blotting in human cell lines. https://www.cellsignal.com/products/primary-antibodies/rhoe-4-mouse-mab/3664<br>Rabbit monoclonal anti-RNA Polymerase II (D8L4Y), Cell Signaling was validated in human cell lines for ChIP. https://www.cellsignal.com/products/rpb1-ntd-d8l4y-rabbit-mab/14958<br>Mouse monoclonal anti-HA (66006-2-Ig), Protein Tech was validated in human cell line for Immunoprecipitation. https://www.ptglab.com/products/HA-Tag-Antibody-66006-2-Ig.htm<br>Rabbit monoclonal anti-p21 (12D1) , Cell Signaling was validated in a human cell line for immunofluorescence staining. https://www.cellsignal.com/products/primary-antibodies/p21-waf1-cip1-12d1-rabbit-mab/2947<br>Rabbit monoclonal anti-MYC (D84C12), Cell Signaling was validated for western blotting in a human cell line. https://www.cellsignal.com/products/primary-antibodies/c-myc-d84c12-rabbit-mab/5605<br>Rabbit monoclonal anti-DNMT1 (D63A6), Cell Signaling was validated for western blotting in a human cell line. https://www.cellsignal.com/products/primary-antibodies/dnmt1-d63a6-xp-rabbit-mab/5032<br>Rabbit monoclonal anti-HA (C29F4), Cell Signaling was validated for western blotting in human cell line. https://www.cellsignal.com/products/primary-antibodies/ha-tag-c29f4-rabbit-mab/3724<br>Rabbit monoclonal anti-CDK9 (C12F7), Cell Signaling was validated for western blotting in human cell lines. https://www.cellsignal.com/products/primary-antibodies/cdk9-c12f7-rabbit-mab/2316<br>Rabbit polyclonal anti-PhosphoThr186 CDK9, Cell Signaling was validated for western blotting in human cell lines. https://www.cellsignal.com/products/primary-antibodies/phospho-cdk9-thr186-antibody/2549<br>Mouse monoclonal anti-IGG (G3A1), Cell Signaling was validated for immunoprecipitation in human cell lines. https://www.cellsignal.com/products/primary-antibodies/mouse-g3a1-mab-igg1-isotype-control/5415

Eukaryotic cell lines

Policy information about cell lines

Cell line source(s)	Primary human keratinocytes were used to generate data for all cell-based experiments. No experimental data and conclusions in this research were derived from cell lines.<br>3T3 cells were used only as feeder cells for clonogenicity assays.<br>293T cells and phoenix cells were only used to generate lentivirus and retrovirus for gene transfer to human keratinocytes. These cell lines were generous gifts from Khavari Lab at Stanford University. These cell lines are also commercially available from ATCC (CRL-3213, CRL-3216, CRL-1658).
Authentication	Cell lines were authenticated by morphology.
Mycoplasma contamination	All cell lines and primary human keratinocytes were tested negative, using "MycoFluor Mycoplasma Detection Kit" (cat#M7006) from ThermoFisher
Commonly misidentified lines<br>(See ICLAC register)	No commonly misidentified cell lines were used.

Human research participants

Policy information about studies involving human research participants

Population characteristics	Although primary human keratinocytes were used, this research was reviewed by Northwestern University Institutional Review Board (IRB) and assigned a determination of NOT HUMAN RESEARCH. The keratinocytes were pooled from minimally 3-6 independent donors. Because the keratinocytes were mixed from different donors and because the sequencing data

	(RNA-seq and ChIP-seq) only covers a very small proportion of the genome, it is impossible to associate the genomic data with any distinct individuals.
Recruitment	not applicable.
Ethics oversight	*Identify the organization(s) that approved the study protocol.*

Note that full information on the approval of the study protocol must also be provided in the manuscript.

ChIP-seq

Data deposition

☒ Confirm that both raw and final processed data have been deposited in a public database such as GEO.

☒ Confirm that you have deposited or provided access to graph files (e.g. BED files) for the called peaks.

Data access links<br>*May remain private before publication.*	https://www.ncbi.nlm.nih.gov/geo/query/acc.cgi?acc=GSE182959
Files in database submission	GSM5543720 ChIP_AFF1_UD<br>GSM5543721 ChIP_AFF1_UD_2<br>GSM5543722 ChIP_HEXIM1_UD<br>GSM6181364 HACDK9_DMSO<br>GSM6181365 HACDK9_DMSO_r2<br>GSM6181366 HACDK9_KL2<br>GSM6181367 HACDK9_KL2_r2<br>GSM6181368 HACDK9_TPA<br>GSM6181369 HACDK9_TPA_r2<br>GSM6181370 ChIP_HEXIM1_UD_r2<br>GSM6181371 PolII_DF1<br>GSM6181372 PolII_DF2<br>GSM6181373 PolII_DMSO_1hrA<br>GSM6181374 PolII_DMSO_1hrB<br>GSM6181375 PolII_DMSO_3hr<br>GSM6181376 PolII_DMSO_3hr_r2<br>GSM6181377 PolII_KL2_1hrA<br>GSM6181378 PolII_KL2_1hrB<br>GSM6181379 PolII_KL2_3hr<br>GSM6181380 PolII_KL2_3hr_r2<br>GSM6181381 PolII_TPA_1hr<br>GSM6181382 PolII_TPA_1hr_r2<br>GSM6181383 PolII_TPA_30minA<br>GSM6181384 PolII_TPA_30minB<br>GSM6181385 PolII_UD1<br>GSM6181386 PolII_UD2
Genome browser session<br>(e.g. UCSC)	https://genome.ucsc.edu/s/sarahlloyd2022/All_Revisions

Methodology

Replicates	2 biological replicates were completed
Sequencing depth	~20M reads passing MAPQ score of 30
Antibodies	Rabbit anti-RNA Polymerase II, Cell Signaling #14958<br>mouse monoclonal HA, Protein Tech Cat#66006<br>Rabbit anti-AFF1 serum, Shilatifard lab<br>Rabbit polyclonal anti-HEXIM1, Abcam, Cat#ab25388
Peak calling parameters	MACS2 with p 0.01
Data quality	MAPQ score minimum of 30, p value for peak calling < 0.01
Software	Bowtie2 for alignment, MACS2 for peak calling
